# Lysionotin exerts antinociceptive effects in various models of nociception induction

**DOI:** 10.1016/j.heliyon.2023.e15619

**Published:** 2023-04-18

**Authors:** Abdelrahim Alqudah, Esam Y. Qnais, Mohammed A. Wedyan, Hakam AlKhateeb, Shtaywy S. Abdalla, Omar Gammoh, Mohammad A. AlQudah

**Affiliations:** a Department of Clinical Pharmacy and Pharmacy Practice, Faculty of Pharmaceutical Sciences, The Hashemite University, Zarqa, Jordan; b Department of Biology and Biotechnology, Faculty of Science, The Hashemite University, Zarqa, Jordan; c Department of Basic Medical Sciences, Faculty of Medicine, Yarmouk University, Irbid, Jordan; d Department of Biological Sciences, Faculty of Science, University of Jordan, Amman, Jordan; e Department of Clinical Pharmacy and Pharmacy Practice, Faculty of Pharmacy, Yarmouk University, Irbid, Jordan; f Department of Physiology, Jordan University of Science and Technology, Irbid, Jordan

**Keywords:** Lysionotin, Nociception, Flavonoids, Naloxone, Vanilloid, Glutamate

## Abstract

**Background:**

Lysionotin, a natural flavonoid extracted from *Lysionotus pauciflorus* Maxim (Gesneriaceae), has several pharmacological effects including anti-bacterial, anti-hypertensive and anti-inflammatory effects. However, its analgesic effect has not been investigated. This study aimed to assess the antinociceptive activity of lysionotin using chemically and thermally induced nociception in a mouse model.

**Methods:**

The antinociceptive effects of various lysionotin doses (50, 100, 150, and 200 μg/kg) were assessed in mice using the acetic acid-induced writhing test, hot plate test, and formalin-induced paw licking assay. The effects were compared to those of mice treated with acetylsalicylic acid or morphine in the presence or absence of naloxone (an opioid receptor antagonist). Capsaicin- and glutamate-induced paw licking tests were also used to evaluate the involvement of the vanilloid and glutamatergic systems, respectively.

**Results:**

Lysionotin produced significant dose-dependent inhibition of nociceptive behavior in the acetic acid-induced writhing test, showing 60% inhibition at a dose of 200 μg/kg. Lysionotin also caused a significant increase in the latency period in response to the hot plate test (76.4% at 200 μg/kg), and significantly inhibited both the neurogenic and inflammatory phases in the formalin-induced paw licking test. Naloxone significantly reverses the effect of lysionotin in both hot plate test and formalin-induced paw licking test. Moreover, lysionotin significantly inhibited the neurogenic nociception induced by intraplantar injections of glutamate and capsaicin (57% and 67.2%, respectively at a dose of 200 μg/kg). Thus, lysionotin exhibited peripheral and central antinociception through the modulation of vanilloid receptors, opioid receptors, and the glutamatergic system.

**Conclusion:**

Lysionotin possesses antinociceptive activity on adult mice that is mediated through both central and peripheral pathways.

## 1 Introduction

Pain is defined as an unpleasant sensation or feeling that is caused by damage to body tissues or is associated with damage [1]. The general scientific consensus is that pain, whether due to actual or potential tissue damage, serves to protect the organism from injury [2,3]. Nociceptive pain is one of the two major types of pain caused by damaging stimuli to the body tissues. Nociceptive pain usually occurs when nociceptors are activated by strong cold, intense heat, harsh mechanical stimuli, or a variety of chemical stimuli. In the last few decades, significant attention has been paid to the underlying mechanisms of pain [4]. Moreover, the neurotransmitter systems, neuromodulators, ion channels, and receptors involved in pain neural pathways have been exhaustively reviewed [5,6].

Pain decreases the quality of life, decreases productivity, increases absenteeism from work, causes disability, and may cause unemployment in a significant sector (up to one-third) of the population. The yearly cost of such encounters is estimated to be billions of dollars [7]. Regardless of the high cost of treatment, the individual response of patients to analgesics cannot be predicted and may fall short of the intended pain relief strategy. This may be because many drugs have side effects that discourage patient compliance with physicians' recommendations.

Non-steroidal anti-inflammatory drugs (NSAIDs) are considered one of the most frequently used therapeutics that can be used to treat inflammatory-related pain. Although NSAIDs, their long-term use is associated with several significant adverse effects such as bleeding, peptic ulcers, and gastrointestinal lesions [8].

Likewise, opioids are another major class of analgesics used in patients with severe pain which is prescribed for short term post operative/neurogenic pain. Opioids have significant side effects such as tolerance, respiratory depression, and constipation [9]. Therefore, alternative therapeutic agents with mild action and fewer side effects (such as plant-based medicines) are receiving increasing attention.

The antinociceptive effects of any compound can be assessed using several models. Acetic acid-induced writhing test directly activates a non-selective ion channel in peripheral nociceptive fibers [10]. It also acts indirectly by releasing several autacoid mediators—such as serotonin, histamine, bradykinin, and substance P—and increasing the levels of prostaglandins (E2 and F2). Moreover, acetic acid triggers the production of lipoxygenase, and eventually activates peripheral nociceptive neurons [11]. In addition, the hot plate test is also used during which the paw licking and jumping behavior in response to hot plate stimulation occurs at the supraspinal level, and the duration of the jumping and licking can be used to assess animal responses [12]. Moreover, the latency time of mice in the hot plate test can be prolonged only by centrally acting opioid-like drugs, but not peripherally acting drugs. Moreover, the formalin-induced paw-licking test which has two discrete phases of nociceptive behavior was observed after formalin injection. The first phase started immediately after the injection of formalin, and continued for approximately 5 min, whereas the second phase occurred 15–30 min after the administration of formalin. The first phase is a neurogenic phase that is initiated by the direct action of formalin on pain receptors, whereas the second phase is the inflammatory phase that is caused by the release of inflammatory mediators, such as histamine, prostaglandins, and bradykinins [[13], [14], [15]]. Furthermore, the capsaicin-induced paw-licking test induces both hyperalgesia and analgesia, and it is therefore used in pain research [16,17]. Capsaicin stimulates TRPV1 receptors, causes the influx of Ca^2+^ and Na^+^ (mostly Ca^2+^), and thus activates C- or Aδ-fibers in afferent neurons, which leads to neurogenic pain. Moreover, a glutamate-induced paw-licking test is also used. Glutamate is an important excitatory amino acid and neurotransmitter. It is present at high levels in the central nervous system and is essential for myriad physiological and pathophysiological conditions [18]. Glutamate receptors are distributed in the central and peripheral nervous systems, and their activation leads to nociceptive transmission.

Flavonoids have attracted much interest among researchers because they exist widely in plants and exhibit several significant pharmacological activities. Flavonoids have been widely shown to play a role in antinociception and inflammation [[19], [20], [21], [22], [23]]. Many flavonoids have been demonstrated to bind opioid and GABA_A_ receptors to produce potent antinociception in different pain models. This analgesic effect may occur directly through the inhibition of the afferent pathways in the periphery, or indirectly by increasing traffic through the descending pathways [24].

Lysionotin (5,7-dihydroxy-4,6,8-methoxyflavone) is a naturally occurring flavonoid commonly present in different herbs such as *Lysionotus pauciflorus* Maxim and *Ocimum basilicum* [25]. Regarding its chemical structure, lysionotin is a trimethoxy compound that belongs to the flavones family of flavonoids [25]. It has been shown to exert antibacterial effects by reducing the expression of α-toxin, which is necessary for *Staphylococcus aureus* pathogenicity [26]. Moreover, lysionotin also performs free radical scavenging activities and has anti-hypertensive effects [21]. Furthermore, lysionotin demonstrates an anti-inflammatory effect in a carrageenan-induced rat model of paw oedema [27].

Several review articles in literature have emphasized that flavonoids, in general, can exert effective analgesic activities for both acute and chronic pain management through different acting mechanisms [[28], [29], [30]]. Additionally, several flavonoids that have similar chemical structure, trimethoxy, to lysionotin and belong to the same flavonoid family, flavones, of lysionotin, were found to have a strong antinociceptive activity on mice [31,32]. Thus, it is hypothesized that lysionotin could possess antinociceptive effects similar to other trimethoxyflavones.

Until now, there has been no study to investigate the antinociceptive activity of lysionotin. Therefore, this study aims to examine the antinociceptive activity of lysionotin on adult male mice using an Acetic acid-induced writhing test, hot plate test, formalin-induced paw licking test, capsaicin-induced paw licking test and glutamate-induced paw licking test nociception models. Furthermore, it aimed to determine the mechanisms of antinociception exhibited by lysionotin, if present, by applying different nociception behavioral tests.

## 2 Methods

### 2.1 Experimental animals

Adult male Swiss albino mice (weight: 24–28 g) bred and raised in the Animal House Unit at the Hashemite University were used in this study. Approvals for all animal care and experimental procedures were obtained from the Animal Research Ethics Committee at the Hashemite University (IRB number: 34/2020) and were in accordance with the National Institutes of Health Guide for the Care and Use of Laboratory Animals [33]. The mice were maintained under controlled temperature of 21 ± 1 °C with a 12 h light and 12 h darkness schedule (lights on between 0600 and 1800 h). Food and water were provided *ad libitum*. One hour before the experiments, the mice were moved to the testing area for acclimatization to the laboratory conditions to reduce stress. All animal tests were monitored by two trained observers blinded to the experimental design, to avoid bias, in a sound-attenuated room.

### 2.2 Acetic acid-induced writhing test

In this experiment as shown in Table 1, six groups of mice were treated intraperitoneally (i.p.) as described previously [34]. The treatments were as follows: the first group (the vehicle control) received 5% DMSO and 95% distilled water. Lysionotin (A14847, AdooQ Bioscience, USA) was prepared in the vehicle solution to the proper dose and then administered to groups two to five in doses of 50, 100, 150, and 200 μg/kg, respectively. The sixth group was administered 100 mg/kg of acetylsalicylic acid (A5376, Sigma-Aldrich, Merck, USA). Lysionotin doses were chosen based on previous pilot experiments. Acetic acid 0.6% (A6283, Sigma-Aldrich, Merck) was administered in a dose of 10 mL/kg of body weight to each mouse 60 min after the vehicle, lysionotin or acetylsalicylic acid (ASA) treatments. Complete writhing was recorded and counted for 30 min after the acetic acid treatment. Instances of body elongation, abdominal contraction, pelvis ending twisting, and/or trunk twisting associated with limb extension were counted as instances of writhing. The inhibition of writhing was considered the end point of this experiment, and was represented as a percentage according to the following formula:

Percentage of inhibition of writhing (PIW) = [(Writhing number (Control) − writhing number (treatment))/Writhing number (control)] × 100

**Table 1:** Acetic acid-induced writhing test.

	Group (n = 6)	Concentration
1	Vehicle (DMSO)	
2	lysionotin	50 μg/kg
3	lysionotin	100 μg/kg
4	lysionotin	150 μg/kg
5	lysionotin	200 μg/kg
6	Acetylsalicylic acid (ASA)	100mg/kg

### 2.3 Hot plate test

In this test, eight groups of mice were treated as shown in Table 2. Group one (the vehicle control) received 5% DMSO in distilled water. Lysionotin doses (50, 150, 150, and 200 μg/kg) were administered to groups two to five, respectively. Morphine (M8777, Sigma-Aldrich, Merck) was dissolved in sterile saline and administered i.p. (5 mg/kg) to group seven. We tested for the involvement of the opioidergic system in groups six and eight as explained earlier [35]. Group six received the non-selective opioid receptor antagonist, naloxone hydrochloride (5 mg/kg i.p.) (BP548, Merck) just 15 min before treatment with lysionotin (200 μg/kg). Similarly, group eight received naloxone (5 mg/kg i.p.) just 15 min before treatment with morphine (5 mg/kg i.p.). All treatments were administered i.p. 60 min before exposure to testing with the analgesiometer hot plate (55 ± 5 °C). The reaction time was measured as the interval between the instance when the animal was placed on the hot plate and the beginning of paw licking. The reaction time was measured twice; before the treatment and 60 min after the treatment. Results are presented as the percentage increase in baseline according to the following formula: Percentage increase in baseline = ((A − B)/B) × 100, where A represents the reaction time after treatment, and B indicates the reaction time before treatment.

**Table 2:** Hot plate test.

	Group (n = 6)	Concentration
1	Vehicle (DMSO)	
2	Lysionotin	50 μg/kg
3	Lysionotin	100 μg/kg
4	Lysionotin	150 μg/kg
5	Lysionotin	200 μg/kg
6	Naloxone + Lysionotin	5 mg/kg +200 μg/kg
7	Morphine	5 mg/kg
8	Morphine + Naloxone	5 mg/kg+5 mg/kg

### 2.4 Formalin-induced paw licking test

As described before [36], nine groups of mice were treated as follows (Table 3): group one (the vehicle control) received 5% DMSO in distilled water; groups two to five received 50, 100, 150, and 200 μg/kg lysionotin, respectively; group six received morphine (5 mg/kg), and group seven received ASA (100 mg/kg). We also tested for the involvement of the opioidergic system (26) as follows: the nonselective opioid receptor antagonist naloxone hydrochloride (5 mg/kg, i.p.) was administered 15 min before treatment with lysionotin (200 μg/kg; group eight) or morphine (5 mg/kg; group nine). All treatments were administered intraperitoneally. Sixty minutes after treatment, 20 μL of 2.5% formalin (103999, purity 37%, Sigma-Aldrich, Merck) was injected into the subplantar region of the right hind paw to induce pain. The nociceptive response was recorded by measuring the time spent by each mouse licking the formalin injection site. Licking times were recorded in two phases: 0–5 min after formalin injection, representing the early (neurogenic) phase; and 15–30 min after formalin injection, representing the late (inflammatory) phase. The percentage of licking inhibition was calculated using the following formula:

**Table 3:** Formalin-induced paw licking test.

	Group (n = 6)	Concentration
1	Vehicle (DMSO)	
2	Lysionotin	50 μg/kg
3	Lysionotin	100 μg/kg
4	Lysionotin	150 μg/kg
5	Lysionotin	200 μg/kg
6	Naloxone + Lysionotin	5 mg/kg + 200 μg/kg
7	Morphine	5 mg/kg
8	Morphine + Naloxone	5 mg/kg +5 mg/kg
9	Acetylsalicylic acid (ASA)	100 mg/kg

Percentage of inhibition of licking (PIL) = [(Licking time (control) − Licking time (treatment))/Licking time (control)] × 100

### 2.5 Capsaicin-induced paw licking test

This test was performed to evaluate the antinociceptive action of lysionotin through the vanilloid receptor known as Transient Receptor Potential Vanilloid type-1 (TRPV1). As previously described [35], six groups of mice (n = 6 mice/group, Table 4) were treated i.p. as follows: group one (the vehicle control) received 5% DMSO in distilled water, whereas groups two to five received lysionotin doses (50, 100, 150, or 200 μg/kg, respectively). The TRPV1 receptor antagonist known as capsazepine (211280, Sigma-Aldrich, Merck) was administered in a dose of 0.17 mmol/kg i.p. to group six [35]. Sixty minutes after this, 20 μL (1.6 μmol/paw) of capsaicin (211275, Sigma-Aldrich, Merck) was injected via the intraplanar route into the right hind paw of each animal. The nociceptive response by capsaicin was measured as the time each mouse spent licking or biting the capsaicin injection site, and these measurements were recorded 0–5 min after the injection of capsaicin.

**Table 4:** Capsaicin-induced paw licking test.

	Group (n = 6)	Concentration
1	Vehicle (DMSO)	
2	Lysionotin	50 μg/kg
3	Lysionotin	100 μg/kg
4	Lysionotin	150 μg/kg
5	Lysionotin	200 μg/kg
6	Capsazepine	0.17 mmol/kg

### 2.6 Glutamate-induced paw licking test

This test was performed to assess the antinociceptive role of lysionotin through the glutamatergic receptors. As previously described [35,37], five groups of mice (n = 6 mice/group, Table 5) were treated as follows: 5% DMSO was administered i.p. to the first group (the vehicle control), and groups two to five received i.p. treatments of lysionotin (50, 100, 150, 200 μg/kg, respectively). After 60 min, 20 μL of glutamate (1446600, Sigma-Aldrich, Merck) was injected intraplantarly into the ventral surface of the right hind paw of each animal. Immediately after this, the animals were observed for 15 min and the time each mouse spent licking and/or biting the glutamate injection site was measured.

**Table 5:** Glutamate-induced paw licking test.

	Group (n = 6)	Concentration
1	Vehicle (DMSO)	
2	Lysionotin	50 μg/kg
3	Lysionotin	100 μg/kg
4	Lysionotin	150 μg/kg
5	Lysionotin	200 μg/kg

### 2.7 Statistical analysis

The Prism 5 software (GraphPad Software, USA) was used for data analysis. Data are presented as the mean ± standard error of the mean (SEM). Differences between groups were analyzed using one-way ANOVA followed by Tukey’s *post-hoc* test. Statistical significance was set at *P* < 0.05.

## 3 Results

### 3.1 Lysionotin reduced writhing induced by acetic acid

**Fig. 1:**
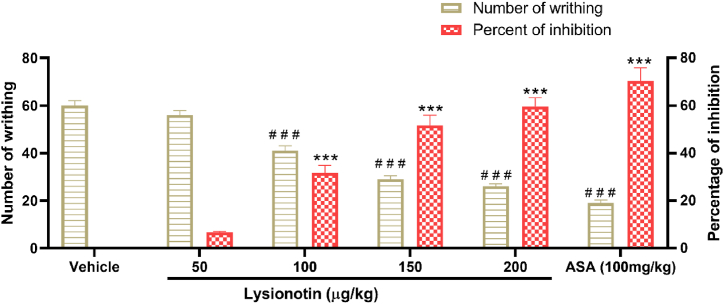
Effect of lysionotin on writhing in mice (n = 6) induced by 0.6% acetic acid. Animals were injected with 5% DMSO (vehicle), lysionotin (50, 100, 150, 200 μg/kg i.p.), or acetylsalicylic acid (ASA, 100mg/kg). Values are the means ± SEM; ***P < 0.001 indicates a significant difference in percent of inhibition compared to the vehicle. ^**# # #**^*P* < 0.001 indicates a significant difference in number of writhing compared to the vehicle. (ANOVA, Tukey’s *post-hoc*).

The i.p. administration of lysionotin (100, 150, 200 μg/kg) led to significant reductions (*P* < 0.001) in the number of acetic acid-induced writhing episodes in treated mice compared to those in the control group (Fig. 1). Additionally, the effect of lysionotin was dose-dependent (*P* < 0.05). The percentage of writhing inhibition reached about 60% in the group that received the highest dose of lysionotin compared to the control group (*P* < 0.001). In contrast, the reduction in nociceptive behavior induced by the reference drug, ASA (100 mg/kg), was about 70% compared to the control group (Fig. 1).

### 3.2 Lysionotin increased latency time in the hot plate test

**Fig. 2:**
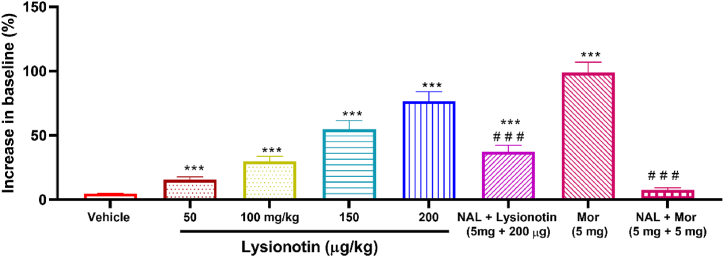
Effect of lysionotin on mice (n = 6) in the hot plate test. Values are the means ± S.E.M. ***P < 0.001 indicates a significant difference compared to the vehicle (5% DMSO). ^# # #^*P* < 0.001 indicates a significant difference compared to 200 μg/kg lysionotin or to 5 mg morphine. (NAL: naloxone, Mor: morphine). (ANOVA, Tukey’s *post-hoc*).

Lysionotin (50, 100, 150, 200 μg/kg) administration led to a significant (*P* < 0.001) increase in the latency time for the animals to lick their posterior paw when placed on a hot plate (Fig. 2). Additionally, the effect of lysionotin was dose-dependent (*P* < 0.05). A lysionotin dose of 200 μg/kg produced the maximum reduction in pain compared to the control group. The percentage increase in the baseline latency time in the lysionotin-treated group (200 μg/kg) was 76.4% (*P* < 0.001). In contrast, morphine, which was used as a reference drug, produced a greater increase in latency time (98.7%).

To assess the mechanism of the antinociceptive effect of lysionotin, we treated the mice with naloxone (an opioid antagonist) 15 min before administering 200 μg/kg of lysionotin or 5 mg/kg of morphine. The mice were then subjected to the hot plate test. The morphine-induced increase in latency time was significantly blocked by treatment with naloxone (*P* < 0.001) compared to morphine treatment alone. Similarly, naloxone significantly blocked the increase in latency time induced by lysionotin (*P* < 0.001) compared to that of lysionotin (200 μg/kg) alone (Fig. 2). However, a fraction of the antinociceptive effect of lysionotin remained unchanged, suggesting that lysionotin uses a different antinociceptive mechanism in addition to the opioidergic system.

### 3.3 Lysionotin reduced licking time in both the early and late phases after formalin

**Fig. 3:**
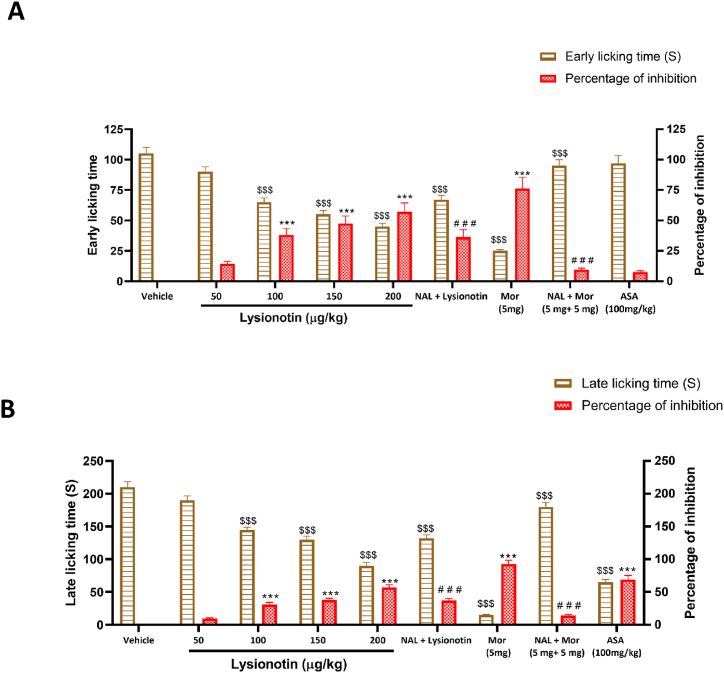
Effect of lysionotin on the early (A) and late (B) phases of 2.5% formalin-induced paw licking behavior in mice (n = 6). Animals were injected with 5% DMSO (vehicle), lysionotin (50, 100, 150, and 200 μg/kg, i.p), morphine (Mor) (5 mg/kg, i.p), or acetyl salicylic acid (ASA, 100 mg/kg, i.p). Naloxone (NAL, 5 mg/kg, i.p.) was administered 15 min before injection with lysionotin (200 μg/kg) or Mor (5 mg/kg, i.p.). Data are the means ± S.E.M. ***P < 0.001 indicates a significant difference in percentage of inhibition compared with vehicle; ^$$$^*P* < 0.001 indicates a significant difference in licking time compared with vehicle. ^# # #^*P* < 0.001 indicates a significant difference compared to the groups treated with lysionotin (200 μg/kg) or morphine (5 mg/kg, i.p.). (ANOVA, Tukey’s *post-hoc*).

Mice treated with 100, 150, and 200 μg/kg of lysionotin showed significant (*P* < 0.001) reductions in licking times in both early and late phases following formalin injection (Fig. 3). The reduction followed a similar pattern in both phases. Similarly, morphine (the reference drug) produced a significant (*P* < 0.001) reduction in licking time in both phases. However, treatment with acetylsalicylic acid failed to decrease the licking time during the early phase (Fig. 3A), although its inhibitory effect in the late phase was significant (*P* < 0.001, Fig. 3B).

To examine the role of the opioidergic system in the antinociceptive effect of lysionotin, naloxone was administered to the mice 15 min before the administration of lysionotin (200 μg/kg) or morphine (5 mg/kg). The animals were then subjected to a formalin-induced paw-licking test. Naloxone prior to morphine significantly (*P* < 0.001) increased the licking time in both early and late phases compared to the licking times in the group treated with morphine alone, and the inhibition percentages were 9.5% and 14.3%, respectively (Fig. 3). Interestingly, when naloxone was administered before 200 μg/kg of lysionotin, the licking times were significantly (*P* < 0.001) reduced in both early and late phases to the same degree (Fig. 3). These results suggested that the effects of lysionotin are mediated by a different mechanism of action in addition to the opioidergic system.

### 3.4 Licking time was reduced with lysionotin treatment after capsaicin injection

**Fig. 4:**
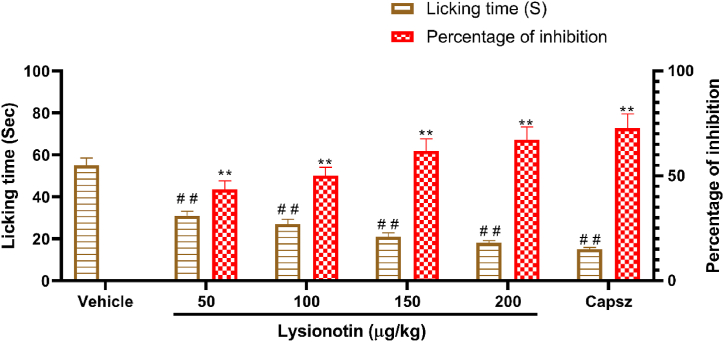
Effect of lysionotin on capsaicin-induced paw licking in mice. Animals were injected with 5% DMSO (vehicle), lysionotin (50, 100, 150, and 200 μg/kg, i.p.), or the capsaicin antagonist, capsazepine (Capsz, 0.17 mmol/kg, i.p.). After 60 min, the mice were challenged with capsaicin, and the licking time was recorded. Each group contained six male mice. Values are the means ± S.E.M. **P < 0.01 indicates a significant difference in percent of inhibition compared to response of the control group. ^# #^*P* < 0.01 indicates a significant difference in licking time compared to response of the control group. (ANOVA, Tukey’s *post-hoc*).

This test was performed to investigate the antinociceptive effect of lysionotin through the vanilloid system. Following, treatment with lysionotin (50, 100, 150 and 200 μg/kg) significantly (*P* < 0.01) reduced the licking time in mice (Fig. 4). This effect of lysionotin was dose-dependent. In addition, treatment with the capsaicin antagonist, capsazepine, significantly (*P* < 0.01) reduced the licking time in mice, and the percentage reduction was 72.7% (Fig. 4).

### 3.5 Lysionotin reduced paw licking time after glutamate injection

**Fig. 5:**
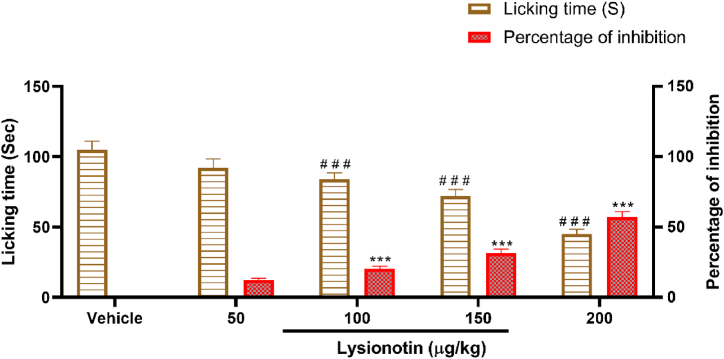
Effect of lysionotin on glutamate-induced paw licking in mice. Animals were injected intraperitoneally with 5% DMSO (vehicle) or lysionotin (50, 100, 150, and 200 μg/kg). After 60 min, the mice were administered 20 μL of glutamate intraplantarly into the right hind paw, and the licking time was recorded. Each group contained six male mice. Values are the means ± SEM. ***P < 0.001 indicates a significant difference in percentage of inhibition compared to the response of the control group; ^# # #^*P* < 0.001 indicates a significant difference in licking time compared to the response of the control group (ANOVA, Tukey’s *post-hoc*).

Lysionotin treatments of 100, 150 and 200 μg/kg significantly (*P* < 0.001) reduced the paw licking time in glutamate-injected mice (Fig. 5). The antinociceptive effect of lysionotin on glutamate injection was dose-dependent (*P* < 0.05).

## 4 Discussion

This study demonstrates, for the first time, the antinociceptive effects of lysionotin. Our results reveal that lysionotin inhibits the well-documented nociceptive effects of acetic acid. Acetic acid-induced nociception involves the production of nitric oxide (NO) [38] and the secretion of several inflammatory cytokines (i.e., TNF-α, IL-1β, and IL-8) from the peritoneal mast cells and macrophages into the peritoneum. This, in turn, activates peripheral nociception receptors [39]. Le Bars et al. (2001) reported that nociception pain was felt very quickly after the i.p. injection of acetic acid. In this study, acetic acid elicited physical responses in the form of writhing [40]. The mechanism of this reflex involves the conversion of arachidonic acid into eicosanoids (essential pain mediators) by lipoxygenases (LOX) and cyclooxygenases (COX). Eicosanoids initiate the release of prostaglandins and other mediators from the peritoneal cavity [41]. Moreover, COX-and LOX-produced eicosanoids cause hyperalgesia by sensitizing the peripheral pain neurons [42]. Nonsteroidal anti-inflammatory drugs such as ASA have been shown to inhibit the synthesis of prostaglandins by blocking the activity of COX and inhibiting the body’s responses to pain [43]. In the present study, lysionotin may have inhibited acetic acid-induced nociception by suppressing the peripheral levels of COX and LOX. This would have indirectly reduced the production of pain mediators (such as prostaglandins), indicating that lysionotin acts as a peripheral antinociceptive agent. However, this speculation should be taken with caution, as other non-analgesic agents—such as antihistamines, anticholinergic agents, and muscle relaxants—often produce false positive results in the acetic acid-induced writhing test [44]. Therefore, we have incorporated other methods (such as formalin and hot plate tests) to determine whether the antinociceptive effects of lysionotin are centrally or peripherally mediated.

The supraspinal and spinal biological properties of novel drugs can be evaluated using the hot plate test without inputs from peripheral nociception neurons [45]. A drug or substance that increases the latency of mice in the hot plate test must have centrally mediated activity, comparable to that of opioids [46,47]. Our results demonstrate that lysionotin prolonged the latency of mice feeling discomfort on the hot plate, suggesting the centrally mediated antinociceptive activity of lysionotin. Considering the results of the acetic acid-induced writhing test, we speculated that lysionotin exerts central and peripheral antinociceptive activity.

The antinociceptive activity of lysionotin at the central and peripheral levels was confirmed using the formalin-induced paw-licking test. Our results showed that lysionotin effectively inhibits nociceptive responses in both phases, confirming its centrally-acting analgesic abilities. This is because centrally acting agents (opioids) are known to inhibit both phases, whereas drugs that act peripherally only inhibit the second phase (e.g., NSAIDs) (29). In addition, pre-treatment of mice with the non-selective opioid antagonist, naloxone, significantly inhibited the antinociceptive effect of morphine, but only partially inhibited the antinociceptive effect of lysionotin in the hot plate test, and the early phase of formalin-induced nociception. These results indicate that, in addition to the opioidergic system, another mechanism may be involved in the antinociceptive action of lysionotin.

To evaluate the ability of lysionotin to modulate pain reception via vanilloid receptors and/or the glutamatergic system, we performed the capsaicin-induced and glutamate-induced paw-licking tests. Capsaicin induces the release of pro-inflammatory peripheral mediators—such as neuropeptides and neurokinins, NO, and excitatory amino acids (glutamate and aspartate)—and can also transduce nociceptive pain from vanilloid receptors to the spinal cord [48]. Moreover, inflammatory mediators activate and sensitize vanilloid receptors, which can also amplify the inflammatory mediator levels, creating a loop to potentiate nociception [16,49]. Our results demonstrated that lysionotin inhibited capsaicin-induced paw licking (Fig. 5). This inhibition was comparable (67.2% vs. 72.7%) to that induced by capsazepine, a known TRPV antagonist. These results indicate that lysionotin effectively interferes with the transmission of nociception through the vanilloid receptors and blocks the release or activity of these inflammatory agents. The latter observation is consistent with the fact that lysionotin can ameliorate the writhing responses and the inflammatory responses in the second phase of the formalin test. Capsazepine also reduced the licking time from approximately 55 s to approximately 15 s, which indicates that capsaicin-induced pain is a valid model for such experiments and verifies the efficacy of the treatment.

Finally, the glutamate-induced paw-liking test was used to investigate the ability of lysionotin to interfere with glutamate-mediated nociceptive transmission. Glutamate injection induced licking behavior in mice. However, the time for this licking behavior was significantly reduced by different concentrations of lysionotin (57% inhibition at 200 μg/kg). This test is similar to the late phase of the formalin test, which was found to be inhibited by glutamate receptor antagonists such as MPEP or CPCCOE. This is in contrast to the neurogenic early phase of the formalin test, which remained unaffected [18,50]. In the present study, the administration of lysionotin inhibited the glutamate-induced nociceptive response, indicating that lysionotin exerts antinociceptive activity in response to glutamate-induced pain activation by acting on the glutamate receptors or by interacting with NO release. However, further investigations are required to elucidate the role of NO and its downstream signaling in the antinociceptive activity of lysionotin.

## 5 Conclusions

Our results demonstrate that the flavonoid, lysionotin, possesses antinociceptive effects that are mediated through a variety of physiological pathways within the peripheral and central nervous systems. It acts as an opioid receptor agonist and can inhibit the vanilloid and glutamatergic receptor systems.

## Author contribution statement

Abdelrahim Alqudah, Esam Y. Qnais, Mohammed A. Wedyan: Conceived and designed the experiments; Wrote the paper.

Hakam AlKhateeb, Shtawy Abdalla: Performed the experiments.

Mohammed Alqudah: Analyzed and interpreted the data.

Omar Gammoh: Contributed reagents, materials, analysis tools or data.

## Data availability statement

Data will be made available on request.

## Additional information

No additional information is available for this paper.

## Ethics approval

All animal procedures were approved by the Animal Research Ethics Committee at the Hashemite University (IRB number: 34/2020) and were in accordance with the guidelines of the U.S. National Institutes of Health on the use and care of laboratory animals and with the Animal Research: Reporting of In Vivo Experiments (ARRIVE) guidelines (https://arriveguid elines.org).

## Funding statement

The animal experiments for this study were funded by a grant from The 10.13039/501100009697Hashemite University, Jordan (Grant # 34/2020).

## Declaration of competing interest

The authors declare that there is no conflict of interest regarding the publication of this article.
